# Oxidative Stress Induced by Chemotherapy: Evaluation of Glutathione and Its Related Antioxidant Enzyme Dynamics in Patients with Colorectal Cancer

**DOI:** 10.3390/nu15245104

**Published:** 2023-12-14

**Authors:** Feng-Fan Chiang, Shih-Chien Huang, Pei-Ting Yu, Te-Hsin Chao, Yi-Chia Huang

**Affiliations:** 1 Division of Colorectal Surgery, Department of Surgery, Taichung Veterans General Hospital, Taichung 40705, Taiwan; hankel.chiang@gmail.com; 2 Department of Food and Nutrition, Providence University, Taichung 43301, Taiwan; 3 Department of Nutrition, Chung Shan Medical University, Taichung 40201, Taiwan; chienchien2011@gmail.com (S.-C.H.); elfjs0928@gmail.com (P.-T.Y.); 4 Department of Nutrition, Chung Shan Medical University Hospital, Taichung 40201, Taiwan; 5 Chiayi & Wanqiao Branch, Taichung Veterans General Hospital, Chiayi 60090, Taiwan; chaotehsin@gmail.com

**Keywords:** glutathione, oxidative stress, antioxidant capacity, chemotherapy, colorectal cancer

## Abstract

One of the mechanisms of chemotherapy is to increase the oxidative stress of cancer cells, leading to their apoptosis. Glutathione (GSH) and its related antioxidant enzymes might be stimulated to cope with increased oxidative stress during chemotherapy. Here, we studied the fluctuation in oxidative stress and GSH-related antioxidant capacities before tumor resection, after tumor resection, and after resection either with or without chemotherapy in patients with colorectal cancer (CRC). This was a cross-sectional and follow-up design. We followed patients before having tumor resection (pre-resection), one month after tumor resection (post-resection), and after the first scheduled chemotherapy (post-chemo). If patients were required to receive chemotherapy after tumor resection, they were assigned to the chemotherapy group. Eligible patients were scheduled to undergo six to twelve cycles of chemotherapy at 2-week intervals and received single, double, or triple chemotherapeutic drugs as required. Those patients who did not require chemotherapy were assigned to the non-chemotherapy group. Indicators of oxidative stress and GSH-related antioxidant capacities were determined at the above three time points. We found in 48 patients of the chemotherapy group and in 43 patients of the non-chemotherapy group different fluctuations in levels of oxidative stress indicators and GSH-related antioxidant capacities starting from pre-resection, post-resection through the post-chemo period. Both groups showed significantly or slightly increased levels of advanced oxidation protein products (AOPP), GSH, and its related enzymes in tumor tissues compared to adjacent normal tissues. Patients in the chemotherapy group had significantly lower plasma levels of GSH and glutathione disulfide (GSSG), but had significantly higher plasma glutathione peroxidase and glutathione reductase activities than patients in the non-chemotherapy group post-chemo. Plasma levels of malondialdehyde and AOPP were positively or negatively associated with GSH and GSSG levels post-chemo after adjustment for age, sex, and histological grading in patients receiving chemotherapy. These significant associations were, however, not seen in patients without chemotherapy. Patients with CRC may require higher GSH demands to cope with a greater oxidative stress resulting from chemotherapy.

## 1. Introduction

Colorectal cancer (CRC) is the third-most frequently diagnosed cancer, with an increasing mortality worldwide in the past decade [1,2]. Resection of colorectal tumors combined with chemotherapy and/or radiation therapy is currently its mainstay curative treatment. Adjuvant chemotherapy (i.e., oxaliplatin plus capecitabine or leucovorin/5-fluorouracil (5-FU)) can improve survival rates of CRC patients previously treated with neoadjuvant chemotherapy or tumor resection [3,4,5,6,7]. However, chemotherapy (single, double, or triplet regimen) likely has potential toxicity and adverse effects, and only approximately 10% to 59% of metastatic CRC patients presented a partial response (PR) or a complete response (CR) after the completion of chemotherapy [8,9,10].

One mechanism of chemotherapy in CRC treatment is to increase oxidative stress in cancer cells by inducing or promoting the generation of reactive oxygen species or their overproduction, leading to apoptosis [8,11,12,13]. Several studies have reported increased levels of oxidative stress and decreased total antioxidant status in patients with small-cell lung cancer after receiving anthracycline-based chemotherapy [14], in bone marrow transplantation patients during their high-dose chemotherapy (that is, busulfan, VP-16, and cyclophosphamide) and radio-chemotherapy (that is, TBI, VP-16, and cyclophosphamide) [15], in cancer patients (i.e., gastric cancer patients receiving 5-FU, adriamyclin and mitomycin; colon cancer patients receiving 5-FU, oxaliplatin, and folinic acid; and prostate cancer patients receiving prednisolone and mitozantrone) two weeks after starting their first dose of chemotherapy when compared with levels a week earlier, and after tumor resection [16], and similarly, in breast cancer patients after receiving chemotherapy (that is, 5-FU, doxorubicin, and cyclophosphamide) compared to before receiving chemotherapy [17] or compared to those patients without chemotherapy [18]. On the other hand, excessively high oxidative stress induced by antineoplastic drugs (i.e., anthracyclines, alkylating agents, platinum coordination complexes, epipodophyllotoxins, and camptothecins) probably interferes with processes of cell replication and reduces the effectiveness of chemotherapy [19]. Anthracyclines (that is, doxorubicin, epirubicin, and daunorubicin) are widely used in single or combined chemotherapy and have been reported to generate the highest levels of oxidative stress, while antifolates and nucleoside and nucleotide analogues produce relatively low levels of oxidative stress [19]. For these reasons, the concomitant use of antioxidant supplementation (i.e., vitamin E, coenzyme Q10, glutathione (GSH)) is considered to restore the depletion of natural antioxidant capacity in the human body during or after chemotherapy [11,13,19].

GSH is the most abundant and essential intracellular antioxidant. It directly neutralizes the superoxide anion radical. Its related antioxidant enzyme, glutathione peroxidase (GPx), is the first line of the antioxidant defense system in the human body [20]. Another related enzyme, glutathione *S*-transferase (GST), detoxifies xenobiotics or products of oxidative stress [20]. During chemotherapy, GSH and its related antioxidant enzymes are likely activated to cope with increasing oxidative stress. The complication occurs when the antioxidant capacity of the human body is overstimulated during such a situation and cancer cells might be protected by chemoresistance, making chemotherapy less effective [21,22]. Review articles have reported that high levels of GSH are independently associated with resistance to chemotherapy and radiation; on the other hand, GSH depletion could improve the susceptibility of cancer cells to various forms of programmed cell death and improve the sensitivity of cancer cells to chemotherapy [23,24]. In an in vitro study, intracellular GSH depletion made cancer cells more sensitive to oxidative stress, overcoming drug resistance and further improving the outcome of cancer therapy [25,26]. However, no significant changes were found in the levels of malondialdehyde (MDA, an indicator of oxidative stress), GSH, and changes in GST activity in CRC patients between before and after 4 weeks of radiotherapy and adjuvant chemotherapy [27]. It remains controversial regarding the exact fluctuations of the oxidant–antioxidant status before and after chemotherapy in CRC patients. To better determine changes in GSH and its related antioxidant enzymes before and after chemotherapy in patients with CRC, we here assessed and compared the fluctuations in oxidative stress and GSH-related antioxidant capacities before tumor resection, after tumor resection, and after chemotherapy in CRC patients either receiving or not receiving chemotherapy.

## 2. Subjects and Methods

### 2.1. Study Design and Sample Size Calculation

**Figure 1:**
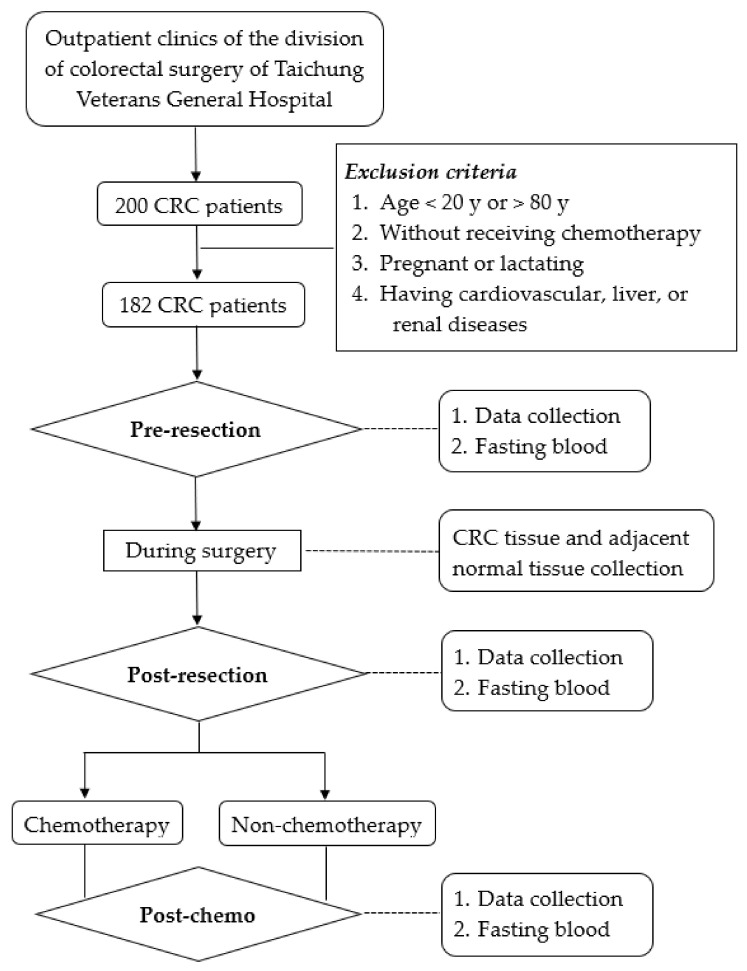
Recruitment and study flow chart.

This was part of our ongoing CRC research study [28]. It had a cross-sectional and follow-up design (Figure 1). We followed patients from the day before receiving tumor resection (pre-resection), one month after tumor resection (the day close to the beginning of chemotherapy, post-resection), and 1 year after tumor resection for patients not receiving chemotherapy or, for patients receiving chemotherapy, when they completed their first scheduled chemotherapy (~1 year after tumor resection, post-chemo).

We calculated the sample size based on the report of Kasapović et al. [17], indicating that the blood GSH level was significantly reduced after receiving chemotherapy compared to before receiving chemotherapy in breast cancer. We assumed a probabilistic 0.5 standardized effect size at a power of 80% with α = 0.05 in a two-sided test. Hence, 34 CRC patients per group were needed to meet the criteria for sample size. We decided to have at least 41 patients given an estimated 20% probability of patients dropping out. Our study was approved by the Institutional Review Board of the Taichung Veterans General Hospital, Taichung, Taiwan (No. CF 19330A, approval date: 30 October 2019).

### 2.2. Subjects

We recruited consecutive patients with colon or rectal cancer (International Classification of Diseases, Tenth Revision, Clinical Modification ICD-10-CM, codes C18–C20, respectively) and with tumor resected from the Colorectal Surgery Division of Taichung Veterans General Hospital, Taiwan. We excluded patients who were <20 or >80 years old, were pregnant or lactating, or had cardiovascular, liver, or kidney disease. Each patient signed an informed consent form prior to participation in the study.

Patients were evaluated for whether they were required to receive or not receive chemotherapy after tumor resection by the colorectal surgeon. Eligible patients were scheduled to undergo 6 to 12 cycles of chemotherapy at 2-week intervals when they had reached stable clinical condition after tumor resection. Eight patients received a single (that is, uracil-tegafur, capecitabine), thirty-five patients received double (that is, 5-FU plus oxaliplatin, 5-FU plus irinotecan, capecitabine plus oxaliplatin), and five patients received triple (that is, 5-FU plus irinotecan plus oxaliplatin) chemotherapeutic drugs as required. These 48 patients were assigned to the chemotherapy group. Other patients who did not require chemotherapy after tumor resection were assigned to the non-chemotherapy group.

Patients with advanced and metastatic CRC cancer (stage IV) were evaluated for their chemotherapy response (that is, CR, PR, stable disease, or progressive disease) after completing their first scheduled chemotherapy. The chemotherapy response was based on the results of computerized tomography and ultrasound scan by a radiologist using the criteria of the Response Evaluation Criteria in Solid Tumors (version 1.1) guidelines [29].

### 2.3. Data Collection and Biochemical Measurements

Data records, blood sampling, tissue collection, and biochemical measurements are briefly described in the following, with more details given in our previous report [28].

We recorded the following data from the patients: age, sex, chemotherapeutic drug use, family history of CRC, diagnosis, staging, histological grading, pathological grading, and tumor location of CRC. Weight was measured pre-resection, post-resection, and post-chemo. Resected colorectal tumor tissue and its adjacent normal tissues were obtained at the time of surgical resection. Fasting blood samples were drawn from patients and stored in vacutainer tubes (Becton Dickinson, Rutherford, NJ, USA) either with or without anticoagulant one day before the tumor resection (pre-resection), one month after the tumor resection for patients not receiving chemotherapy or on a day close to the beginning of chemotherapy for patients receiving chemotherapy (post-resection), and 1 year after tumor resection for patients not receiving chemotherapy or within 2 weeks after the completion of the first scheduled chemotherapy for patients receiving chemotherapy (approximately 1 year after tumor resection, post-chemo).

The Department of Pathology and Laboratory Medicine of Taichung Veterans General Hospital was responsible for analyzing the following: serum levels of albumin, C-reactive protein (CRP), alanine and aspartate aminotransferase (ALT and AST), creatinine, carcinoembryonic antigen (CEA), and carbohydrate antigen 19-9 (CA 19-9). The standard reference serum tumor marker levels for CEA and CA 19-9 were <5 ng/mL and <35 U/mL, respectively [30]. The laboratory of the corresponding author was responsible for measuring the following: plasma and tissue MDA levels, advanced oxidation protein products (AOPP), GSH, glutathione disulfide (GSSG), glutathione reductase (GR), GPx, GST, and trolox equivalent antioxidant capacity (TEAC). Levels of MDA and AOPP were used to reflect oxidative stress, while levels of GSH, GSSG, and TEAC and the activities of GR, GPx, and GST were used to reflect antioxidative capacity. Methods of analyses and brands of commercial kits are not described here, as details are reported in our previous study [28].

### 2.4. Statistical Analysis

Analyses were performed using the SAS statistical software package (version 9.4; Statistical Analysis System Institute Inc., Cary, NC, USA). The normality of the sample distribution was tested using the Shapiro–Wilk test. Demographic, clinical, and biochemical parameters were compared between groups using either Student’s *t*-test or the Mann–Whitney rank sum test. Friedman repeated measures analysis of variance on rank was performed to compare differences among levels pre-resection, post-resection, and post-chemo within the group. Chi-square or Fisher’s exact tests were used in analyzing categorical variables. Parameter differences between tumors and their adjacent normal tissue were compared using the paired *t*-test or the Wilcoxon signed rank test. Partial Spearman correlation was used to assess the relationship between oxidative stress indicators (MDA and AOPP) and both the GSH level and its related antioxidant capacities after adjustment for age, sex, and histological grading. Values are presented as mean ± standard error, and categorical variables as counts and percentages. The results were considered statistically significant at two-tailed *p* < 0.05.

## 3. Results

Although 182 CRC patients participated in this study, only 48 of them had received chemotherapy (chemotherapy group) and 43 patients not receiving chemotherapy had completed the 1 year follow-up (non-chemotherapy group). The responses of 16 stage IV patients to chemotherapy were evaluated after the first run of chemotherapy; one patient had a PR condition, fourteen patients were in the SD condition, and one patient was in the PD condition. Table 1 shows the demographic and clinical characteristics of CRC patients before resection, after resection, and after chemotherapy. Patients in both groups significantly lost weight right after tumor resection, but their weight gradually returned to their pre-resection levels. Although patients receiving chemotherapy had significantly higher serum levels of ALT, AST, and creatinine after completing chemotherapy compared to levels before and after tumor resection, these levels were within the normal range. There were no significant changes in serum levels of ALT, AST, creatinine, and CRP in non-chemotherapy patients at all three different times. Patients in the chemotherapy group had a mean value of CEA and CA19-9 tumor markers below the reference value (<5 ng/mL for CEA and <35 U/mL for CA19-9) after receiving chemotherapy.

Plasma biochemical parameters in both groups at three different times are presented in Table 2. Patients in the two groups showed different fluctuation patterns in terms of levels of oxidative stress indicators and GSH-related antioxidant capacities at all three time points. Patients in the chemotherapy group showed significantly or slightly higher levels of MDA, GPx, GR, GST, and TEAC in the post-chemo period compared to the pre- and post-resection periods. In contrast, the non-chemotherapy group showed either unchanged or slightly lower levels of AOPP, GSH, GPx, GR, GST, and TEAC in the post-chemo period compared to the pre- and post-resection periods. Patients receiving chemotherapy showed significantly higher levels of plasma GSH and GSSG after tumor resection compared to pre-resection, and these levels dropped to levels close to pre-resection in the post-chemo period. On the other hand, patients in the non-chemotherapy group had the plasma GSH level unchanged throughout the three different time points and gradually showed an increased plasma GSSG level from pre-resection, post-resection, through the post-chemo period. Patients in the chemotherapy and non-chemotherapy groups showed similar levels of oxidative stress indicators and GSH-related antioxidant capacities in both the pre- and post-resection periods. However, post-chemo, patients in the chemotherapy group had significantly lower levels of plasma GSH and GSSG, but had significantly higher plasma GPx and GR levels than patients in the non-chemotherapy group.

Table 3 shows the levels of oxidative stress indicators, GSH, and its related antioxidant capacities in tumor tissue and its adjacent normal tissue. In the tumor tissues of both groups, we found significantly or slightly increased levels of AOPP and GSH and their related enzyme activities when compared to adjacent normal tissues.

We computed the partial Spearman correlation coefficients to see whether indicators of oxidative stress (MDA and AOPP) were associated with GSH and its related antioxidant capacities in CRC patients receiving or not receiving chemotherapy. The results are listed in Table 4. Histological grading (degree of tumor differentiation) likely had an influence on oxidative stress, after this confounding factor as well as age and sex had been adjusted. In the chemotherapy group, plasma MDA and AOPP levels after chemo treatment were positively or negatively associated with GSH and GSSG levels post-chemo, after adjustment for age, sex, and histological grading. In contrast, such significant associations were not found in the non-chemotherapy group.

## 4. Discussion

Chemotherapeutic drugs directly or indirectly increase oxidative stress, leading to apoptosis of cancer cells [8,11,12,13]. Cancer patients undergoing chemotherapy might deplete their antioxidant materials or enzymes to overcome the increased oxidative stress. We measured MDA and AOPP levels as biological markers of oxidative stress for CRC patients. Interestingly, the plasma MDA and AOPP levels post-chemo, as well as their association with GSH and GSSG, were opposite. MDA is the final product of lipid peroxidation; its level in patients with CRC can differentiate tumor invasion depth or the presence of lymph node metastasis [31]. MDA-DNA complexes are pro-mutagenic, inducing gene mutations in human tumors [32]. We speculate that, in response to chemotherapy, the MDA level is likely more sensitive than the AOPP level. Greater oxidative stress (plasma lipid hydroperoxides) and reduced plasma levels of GSH, GPx, and GR are reported in breast cancer patients after chemotherapy [17]. Although we also observed that plasma GSH and GSSG levels were both lowered in our CRC patients after chemotherapy compared to non-chemotherapy patients, their plasma GPx and GR levels in response to chemotherapy were increased instead of decreased. GSH is an important antioxidant nutrient in the human body due to its active sulfhydryl group (-SH), which serves as a coenzyme for GPx, GR, and GST. We assumed that after chemotherapy, GSH might be exhausted to support its related antioxidant enzyme activities to cope with increased oxidative stress during chemotherapy, and plasma GSH level would possibly return to the pre-chemotherapy level at a slower rate than its related antioxidant enzymes. Unfortunately, we did not follow our patients for a longer period after completing their first scheduled chemotherapy. Therefore, further changes in their plasma GSH level were not able to be determined. On the other hand, erythrocyte MDA, GSH, and GST levels determined 4 weeks after adjuvant chemotherapy appeared similar to initial levels before the start of combined chemotherapy and radiotherapy [27]. Koçer et al. [33], 6 weeks after chemotherapy, found similar erythrocyte levels of MDA, GSH, and GPx between CRC patients with and without receiving 5-FU treatment. The fluctuations of oxidative stress and GSH-related antioxidant capacities in response to the chemotherapy process need to be further investigated in CRC patients.

Regardless of the inconsistent results between our study and the literature, we wonder whether fluctuations in GSH and its related antioxidant capacity before and after chemotherapy could have an impact on the response to chemotherapy. Sharma et al. [34] reported a drop in the plasma level of MDA, while plasma antioxidant levels (i.e., catalase, superoxide dismutase, GSH, GPx, and GST) increased significantly in advanced cervical cancer patients who had a CR condition compared to those patients with PR or non-response after chemotherapy. Identically, Lu et al. [35] found that gastric patients in the response group (CR plus PR) had significantly higher levels of antioxidant nutrients and enzymes (i.e., GSH, catalase, superoxide dismutase) but decreased MDA levels compared with patients in the non-response group after the completion of two cycles and four cycles of neoadjuvant chemotherapy. Although we did not intend to assess the effect of GSH and its related antioxidant enzyme activities on the chemotherapy response, we speculated that the drop in both plasma GSH and GSSG levels and a rise in antioxidant enzyme levels might be due to the rehabilitated equilibrium of oxidants–antioxidants after the death of tumor cells or the arrest of tumor growth due to chemotherapy. If further studies could be conducted to verify this speculation, along with cumulative evidence showing the role of GSH during cancer chemotherapy [23,24,25,26], the levels of GSH or its related antioxidant enzymes before chemotherapy could be considered a prognostic marker of the response to chemotherapy. However, it may be too early to give any clinical proposal on how to deal with the increase or decrease in the level of GSH before cancer patients receive chemotherapy at this point.

In agreement with previous studies [20,36,37,38,39,40], including ours [28], tumor cells likely take advantage of GSH and its related antioxidant enzymes to protect themselves against increased oxidative stress. Hence, we found that the colorectal tumor tissue had both higher oxidative stress and higher GSH-related antioxidant enzyme activities than its adjacent normal tissue in our CRC patients regardless of chemotherapy. Zaidieh et al. [41] stated that cancer cells with a high baseline reactive oxygen species are more resistant to chemotherapeutic drugs (that is, cisplatin and dequalinium) compared to those cells having fewer reactive oxygen species. Our CRC patients receiving chemotherapy had lower levels of AOPP, GSH, and GSSG in tumor tissues than did non-chemotherapy patients. We speculated that these patients likely had a better response to chemotherapy. Unfortunately, this speculation had not been verified in the present study.

In our study, we not only analyzed plasma samples before and after tumor resection and after chemotherapy, but we also analyzed tumor tissues and adjacent normal tissues. However, only 16 stage IV patients who underwent chemotherapy were evaluated for their chemotherapy response. Such a small sample size did not provide enough statistical power to reveal a clear association between oxidant–antioxidant status and chemotherapeutic response. Furthermore, if oxidant and antioxidant levels were also determined in erythrocytes, we could better understand the changes in oxidant and antioxidant indicators in the circulation and tissue before and after chemotherapy.

## 5. Conclusions

Patients in the chemotherapy and non-chemotherapy groups showed different fluctuation patterns in their levels of oxidative stress indicators and GSH-related antioxidant capacities at three different time points: pre-resection, post-resection, and post-chemo. Patients with CRC likely had higher GSH demands in order to cope with increased oxidative stress during chemotherapy.

## Figures and Tables

**Table 1:** Demographic and clinical characteristics of patients with colorectal cancer at three different times.

Parameters	Chemotherapy (n = 48)			Non-Chemotherapy (n = 43)		
	Pre-Resection	Post-Resection	Post-Chemo	Pre-Resection	Post-Resection	Post-Chemo
Age (y)	59.33 ± 1.62			59.12 ± 1.71		
Male/Female	26/22			23/20		
Weight (kg)	63.41 ± 2.42 ^a^	60.15 ± 2.45 ^b^	64.72 ± 2.53 ^a^	63.96 ± 1.86 ^a^	61.99 ± 1.89 ^b^	65.46 ± 2.04 ^a^
Serum ALT (U/L)	20.19 ± 1.90 ^b^	23.52 ± 2.31 ^b^	29.96 ± 3.31 ^a,†^	23.29 ± 3.87	30.70 ± 6.98	23.07 ± 2.69
Serum AST (U/L)	23.50 ± 1.78 ^b^	25.25 ± 1.85 ^b^	32.08 ± 1.91 ^a,†^	22.20 ± 2.05	23.05 ± 2.10	24.23 ± 1.96
Serum creatinine (mg/dL)	0.85 ± 0.03 ^b^	0.83 ± 0.04 ^b,^**	0.92 ± 0.04 ^a^	0.89 ± 0.04 ^b^	0.93 ± 0.04 ^a,b^	0.93 ± 0.04 ^a^
C-reactive protein (mg/dL)	1.08 ± 0.30 ^a,b^	1.46 ± 0.39 ^b^	0.37 ± 0.17 ^a^	0.80 ± 0.22	0.68 ± 0.14	0.33 ± 0.14
Serum albumin (g/dL)	4.07 ± 0.06 ^b^	4.17 ± 0.05 ^b^	4.32 ± 0.05 ^a,†^	4.13 ± 0.08 ^c^	4.30 ± 0.06 ^b^	4.45 ± 0.05 ^a^
CEA (ng/mL)	77.35 ± 47.57 ^b^	83.75 ± 61.52 ^a^	4.36 ± 1.03 ^a^	27.33 ± 11.33	10.09 ± 5.09	7.26 ± 3.18
CA 19-9 (U/mL)	288.93 ± 263.34 ^a^	124.0 ± 95.25 ^b^	18.01 ± 4.30 ^a^	73.59 ± 54.67	17.56 ± 5.53	17.35 ± 3.52
Stage at diagnosis (n, %) Stage 0 Stage I Stage II Stage III Stage IV	– 0 0 32, 66.7% 16, 33.3%			3, 7% 8, 18.6% 11, 25.6% 13, 30.2% 8, 18.6%		
Tumor location (n, %) Ascending colon Transverse colon Descending colon Sigmoid colon Rectum	10, 20.8% 1, 2.1% 8, 16.7% 14, 29.2% 15, 31.3%			8, 18.6% 1, 2.3% 3, 6.3% 11, 22.9% 20, 41.7%		
Concurrent chemoradiotherapy before tumor resection (n, %)	8, 16.7%			–		
Histological grading (n, %) Well-differentiated Moderately differentiated Moderately to poorly differentiated Poorly differentiated	1, 2.1% 40, 86.3% 2, 4.2% 5, 10.4%			1, 2.3% 41, 95.3% 0 1, 2.3%		
Chemotherapy type (n, %) Single therapy Double therapy Triple therapy	8, 16.7% 35, 72.9% 5, 10.4%			– – –		

Values are means ± standard error of the mean. ALT, alanine aminotransferase; AST, aspartate aminotransferase; CRP, C-reactive protein; CEA, carcinoembryonic antigen; CA 19-9, carbohydrate antigen 19-9. ^a,b,c^ Values with different superscript letters are significantly different among pre-resection, post-resection, and post-chemo within the group; *p* < 0.05. ** Value is significantly different between groups post-resection; *p* < 0.05. ^†^ Value is significantly different between groups post-chemo; *p* < 0.05.

**Table 2:** Plasma glutathione and its related antioxidant capacities and indicators of oxidative stress in patients with colorectal cancer at three different times.

Parameters	Chemotherapy (n = 48)			Non-Chemotherapy (n = 43)		
	Pre-Resection	Post-Resection	Post-Chemo	Pre-Resection	Post-Resection	Post-Chemo
MDA (μmol/L)	1.0 ± 0.03 ^b^	1.05 ± 0.04 ^b^	1.28 ± 0.05 ^a^	0.97 ± 0.03 ^b^	1.06 ± 0.05 ^a,b^	1.22 ± 0.06 ^a^
AOPP (μmol/L)	380.67 ± 20.77 ^b^	523.0 ± 36.71 ^a^	463.64 ± 30.28 ^a,b^	385.73 ± 28.82	444.41 ± 35.77	460.96 ± 30.85
GSH (μmol/L)	55.74 ± 4.66 ^a^	89.69 ± 8.14 ^b^	54.87 ± 4.81 ^a,†^	73.47 ± 8.52	97.07 ± 8.06	79.78 ± 5.19
GSSG (μmol/L)	429.34 ± 13.24 ^c,^*	522.97 ± 14.76 ^b^	458.14 ± 15.18 ^a,†^	468.78 ± 12.64 ^b^	552.20 ± 12.64 ^a^	585.48 ± 13.21 ^a^
GPx (nmol/mL/min)	193.01 ± 10.07 ^c,^*	238.16 ± 9.65 ^b^	275.92 ± 10.15 ^a,†^	159.51 ± 8.62 ^b^	232.19 ± 9.93 ^a^	232.53 ± 11.01 ^a^
GR (nmol/mL/min)	58.50 ± 2.79 ^b^	69.74 ± 3.01 ^a^	73.88 ± 2.43 ^a,†^	57.17 ± 3.67	63.93 ± 2.31	64.87 ± 3.04
GST (nmol/mL/min)	16.91 ± 2.49 ^b^	26.19 ± 2.93 ^a^	28.27 ± 4.25 ^a^	19.54 ± 2.26 ^b^	32.75 ± 3.82 ^a^	23.81 ± 2.81 ^a,b^
TEAC (μmol/L)	3811.91 ± 53.52 ^b^	4382.70 ± 53.24 ^a^	4461.64 ± 56.79 ^a,†^	3891.11 ± 49.89 ^c^	4428.65 ± 70.91 ^b^	4259.38 ± 54.30 ^a^

Values are means ± standard error of the mean. MDA, malondialdehyde; AOPP, advanced oxidation protein products; GSH, reduced glutathione; GSSG, glutathione disulfide; TEAC, trolox equivalent antioxidant capacity; GPx, glutathione peroxidase; GR, glutathione reductase; GST, glutathione *S*-transferase. ^a,b,c^ Values with different superscript letters are significantly different among pre-resection, post-resection, and post-chemo within the group; *p* < 0.05. * Value is significantly different between groups pre-resection; *p* < 0.05. ^†^ Value is significantly different between groups post-chemo; *p* < 0.05.

**Table 3:** Tissue indicators of oxidative stress, glutathione, and its related antioxidant capacities in patients with colorectal cancer.

Parameters	Chemotherapy (n = 48)		Non-Chemotherapy (n = 43)	
	Tumor Tissue	Adjacent Normal Tissue	Tumor Tissue	Adjacent Normal Tissue
MDA (μmol/g protein)	0.20 ± 0.03	0.23 ± 0.04	0.23 ± 0.05	0.28 ± 0.09
AOPP (μmol/g protein)	109.39 ± 5.60 ^a,^*	89.50 ± 4.47 ^b^	136.27 ± 6.56 ^a^	83.57 ± 4.21 ^b^
GSH (μmol/g protein)	6.91 ± 2.11 *	5.14 ± 0.72 **	11.31 ± 1.41 ^a^	7.27 ± 0.64 ^b^
GSSG (μmol/g protein)	152.65 ± 12.45 ^a,^*	120.18 ± 8.30 ^b,^**	191.94 ± 18.61 ^a^	137.25 ± 6.54 ^b^
GPx (nmol/min/g protein)	117.48 ± 6.34 ^a^	97.97 ± 5.20 ^b^	139.20 ± 9.50 ^a^	77.89 ± 5.25 ^b^
GR (nmol/min/g protein)	129.30 ± 8.94	113.06 ± 7.60	156.32 ± 12.55 ^a^	116.64 ± 8.44 ^b^
GST (nmol/min/g protein)	25.91 ± 1.69 ^a^	21.42 ± 1.68 ^b^	31.70 ± 2.49 ^a^	25.46 ± 1.77 ^b^
TEAC (μmol/g protein)	484.54 ± 32.80	436.24 ± 20.55 **	450.94 ± 21.51 ^a^	363.84 ± 22.77 ^b^

Values are means ± standard error of the mean. MDA, malondialdehyde; AOPP, advanced oxidation protein products; GSH, reduced glutathione; GSSG, glutathione disulfide; TEAC, trolox equivalent antioxidant capacity; GPx, glutathione peroxidase; GR, glutathione reductase; GST, glutathione *S*-transferase. ^a,b^ Values with different superscript letters are significantly different between tumor tissue and adjacent normal tissue within the group; *p* < 0.05. * Value is significantly different in tumor tissue between groups; *p* < 0.05. ** Value is significantly different in normal adjacent tissue between groups; *p* < 0.05.

**Table 4:** Partial Spearman correlation of oxidative stress indicators with glutathione-related antioxidant capacity parameters in patients with colorectal cancer pre-resection, post-resection, and post-chemotherapy.

	MDA (μmol/L)					
	Chemotherapy (n = 48)			Non-Chemotherapy (n = 43)		
	Pre-Resection	Post-Resection	Post-Chemo	Pre-Resection	Post-Resection	Post-Chemo
GSH (μmol/L)	−0.187	−0.041	0.412 **	−0.159	−0.182	−0.184
GSSG (μmol/L)	−0.069	0.014	0.445 **	0.074	−0.074	0.089
GPx (nmol/mL/min)	−0.172	−0.133	−0.087	0.347	−0.069	−0.172
GR (nmol/mL/min)	0.186	0.071	0.042	0.106	0.086	0.106
GST (nmol/mL/min)	−0.168	−0.283	0.202	0.231	0.128	0.052
TEAC (μmol/L)	−0.090	−0.394 **	0.081	0.105	−0.275	0.428 *
	AOPP (μmol/L)					
	Chemotherapy (n = 48)			Non-chemotherapy (n = 43)		
	Pre-resection	Post-resection	Post-chemo	Pre-resection	Post-resection	Post-chemo
GSH (μmol/L)	0.151	0.356 *	−0.323 *	0.435 *	−0.278	−0.167
GSSG (μmol/L)	0.146	0.228	−0.341 *	0.248	−0.497 **	−0.235
GPx (nmol/mL/min)	0.251	0.079	0.249	−0.153	−0.068	−0.009
GR (nmol/mL/min)	0.045	0.208	0.319 *	0.248	0.050	−0.352
GST (nmol/mL/min)	−0.064	0.100	−0.185	0.070	−0.111	−0.132
TEAC (μmol/L)	0.076	0.117	0.211	0.028	0.229	−0.017

Values are *r_s_*, partial Spearman correlation coefficients adjusted for age, sex, and histological grading. MDA, malondialdehyde; AOPP, advanced oxidation protein products; GSH, reduced glutathione; GSSG, glutathione disulfide; GPx, glutathione peroxidase; GR, glutathione reductase; GST, glutathione *S*-transferase; TEAC, trolox equivalent antioxidant capacity. * *p* < 0.05; ** *p* < 0.01.

## Data Availability

The data are contained within the article.

## Abbreviations

The following abbreviations are used in this manuscript:

5-FU	5-fluorouracil
ALT	alanine aminotransferase
AOPP	advanced oxidation protein products
AST	aspartate aminotransferase
CA19-9	carbohydrate antigen 19-9
CEA	carcinoembryonic antigen
CR	complete response
CRC	colorectal cancer
CRP	C-reactive protein
GSH	glutathione
GPx	glutathione peroxidase
GR	glutathione reductase
GSSG	glutathione disulfide
GST	glutathione *S*-transferase
MDA	malondialdehyde
PD	progressive disease
PR	partial response
SD	stable disease
TEAC	trolox equivalent antioxidant capacity
